# Serum cholesterol disturbances in dogs with common endocrinopathies at the time of diagnosis: a retrospective study

**DOI:** 10.1186/s12917-024-04413-0

**Published:** 2025-03-03

**Authors:** WeiChun Huang, Mathieu Victor Paulin, Elisabeth C. R. Snead

**Affiliations:** Internal Medicine Department, Department of Small Animal Clinical Sciences, Western College of Veterinary Medicine, University of Saskatchewan, 52 Campus Drive, Saskatoon, SK S7N 5B2 Canada

**Keywords:** Hypothyroidism, Diabetes mellitus, Hyperadrenocorticism, Hypoadrenocorticism, Dyslipidemia

## Abstract

**Background:**

Although dyslipidemia is commonly reported in dogs, comparative data on the magnitude of serum cholesterol disturbances have not been reported. We aimed to describe the severity of hyper- and hypocholesterolemia in dogs with common endocrinopathies and to evaluate its association with common laboratory parameters. Medical records were reviewed over a decade (2011–2022) for dogs with hypothyroidism, diabetes mellitus (DM), hyperadrenocorticism (HAC), or hypoadrenocorticism (HA), and included signalment, common laboratory and diagnostic imaging parameters, comorbidities, and medications. This retrospective study included 53 dogs with hypothyroidism, 54 with DM, 62 with HAC, and 79 with HA.

**Results:**

Medians [range] of serum cholesterol concentration ([Chol]_s_) for dogs with hypothyroidism, DM, HAC, and HA were 492 [174–1829], 321 [116–928], 309 [151–630], and 112 mg/dL [31–309], and hypercholesterolemia was reported in 91%, 85%, 81%, and 9% for each disorder, respectively. Median [Chol]_s_ was significantly higher in hypothyroid dogs with a serum thyroxine concentration < 0.47 (A = 607) vs. ≥0.47 ug/dL (B = 324 mg/dL) (B-A = -299 mg/dL; 95.21% CI of difference = [-433; -166]; *p* < .0001), and significantly lower in HAC dogs with serum ALP activity < 1,000 U/L (A = 275) vs. ≥1,000 (B = 360 mg/dL) (B-A = + 74 mg/dL; 95.14% CI of difference = [+ 25; +121], *p* = .006). Comparison among all studied endocrinopathies showed that median [Chol]_s_ was significantly higher in hypothyroid dogs and significantly lower in HA dogs, whereas median [Chol]_s_ was similar in HAC and DM dogs.

**Conclusions:**

Serum cholesterol concentration can serve as a valuable tool to suspect certain canine endocrinopathies.

## Background

Dyslipidemia is common in dogs and can be classified as primary or secondary [1, 2]. The term “hyperlipidemia” refers to increased circulating lipids characterized by hypertriglyceridemia and/or hypercholesterolemia. Primary hypercholesterolemia has been reported in several breeds, including Miniature Schnauzer, Beagle, Shetland Sheepdog, Doberman, Rottweiler, Briard, Rough-coated Collie, and Pyrenees Mountain dogs [1, 2]. Secondary causes of persistent fasting hyperlipidemia include endocrine disorders, pancreatitis, hepatic disease, renal disease (e.g., glomerular disease or nephrotic syndrome), and administration of drugs (such as glucocorticoids, phenobarbital, etc.) [1–6]. Dyslipidemia has frequently been described with common canine endocrinopathies including hypothyroidism, diabetes mellitus (DM), hyperadrenocorticism (HAC), and hypoadrenocorticism (HA) [1, 2, 7].

Hypercholesterolemia is frequently reported in dogs with hypothyroidism, due to increases in both low-density lipoproteins (LDL) and high-density lipoproteins (HDL) [3–5]. Poorly controlled insulin-dependant DM (type 1) is associated with hypertriglyceridemia, hypercholesterolemia and increased serum long-chain fatty acids in dogs [8, 9]. The alterations in lipid metabolism in dogs with HAC result from glucocorticoid stimulation of lipolysis which leads to hypercholesterolemia and increased adrenal steroidogenesis [10, 11]. Impaired clearance of LDL along with decreased catabolism of cholesterol secondary to steroid-induced vacuolar hepatopathy and cholestasis may also contribute [3, 12, 13]. Although an opposite effect on lipid metabolism in dogs with HA would be intuitive [14], both hypocholesterolemia and hypercholesterolemia have been reported with HA. In a recent review article compiling data from several case series, hypocholesterolemia was reported in 13% of dogs with “typical” HA and 73% of dogs with “atypical” HA [15], while hypercholesterolemia was reported by Peterson et al.. (1996) in 14% of dogs with “typical” HA [16]. In one human study, lipid profile at the time of diagnosis of Addison’s disease was normal in 82% of patients, and 4% patients had hypercholesterolemia [17].

Canine hyperlipidemia has emerged as an important clinical condition that requires a systematic diagnostic approach and appropriate treatment [2]. The databases MEDLINE [Pubmed] and Science Direct have been searched with the following keywords “dog/canine”, “cholesterol”, “hypocholesterolemia/ hypocholesterolaemia”, “hypercholesterolemia/ hypercholesterolaemia”, “hypothyroidism/hypothyroid”, “diabetes/diabetic”, “hyperadrenocorticism/Cushing”, and “hypoadrenocorticism/Addison” on 08/13/2024; 234 reports were found and reviewed. Based on the literature search, only one study has directly compared serum cholesterol concentrations [Chol]_s_ between multiple endocrinopathies (hypothyroidism and hyperadrenocorticism) [18], but none of them have compared the magnitude of [Chol]_s_ changes across > 2 endocrinopathies. Furthermore, the degree of [Chol]_s_ disturbance has not been described as a means to aid clinicians diagnosing certain endocrinopathies in dogs. A first objective was to report on the prevalence and severity of hyper- or hypocholesterolemia in dogs with common endocrinopathies. A second aim was to compare the prevalence and the severity of hyper- or hypocholesterolemia between different endocrinopathies. We hypothetized that hypocholesterolemia and hypercholesterolemia would be most commonly observed in HA and hypothyroidism, respectively. A third objective was to evaluate the association between [Chol]_s_ and changes in common laboratory measurements. We hypothesized that (a) [Chol]_s_ would be significantly higher in hypothyroid dogs with lower serum thyroxine concentrations ([TT4]_s_); (b) [Chol]_s_ would be significantly higher in DM dogs with higher serum blood glucose; (c) [Chol]_s_ would be significantly higher in HAC dogs with higher serum alkaline phosphate ([ALP]_s_) activity; (d) [Chol]_s_ would be significantly lower in HA dogs with lower serum albumin concentrations ([Alb]_s_).

## Results

### Hypothyroidism

Fifty-three dogs met the inclusion criteria for a diagnosis of hypothyroidism, with a median age of 7 years old (range: 2–15 yo). The most commonly affected breeds included: Labrador Retriever (8/53, 15%), Cocker Spaniel (6/53, 11%), Golden Retriever (3/53, 6%), and Doberman Pinscher (3/53, 6%), and twenty-four other breeds were reported with a prevalence of 2–4%. The hypothyroid group included the following breeds reported to be predisposed to primary hypercholesterolemia: Beagle (*n* = 2), Doberman Pinscher (*n* = 2), Rottweiler (*n* = 2) and Shetland Sheepdog (*n* = 1) [1]. The sex distribution was almost equal, with 26 (49%) male (*n* = 16 neutered and *n* = 10 intact), and 27 (51%) female (*n* = 24 spayed and *n* = 3 intact) dogs. All dogs but one had a diagnosis of primary idiopathic hypothyroidism; the remaining dog was diagnosed with thyroid carcinoma. Comorbidities were present in 13/55 (23%) cases, and included: cardiac diseases (dilated cardiomyopathy [*n* = 2], arrhythmogenic right ventricular cardiomyopathy [*n* = 1]), ocular diseases (idiopathic bilateral anterior uveitis [*n* = 1], cataract [*n* = 1]), idiopathic epilepsy (*n* = 1), laryngeal paralysis (*n* = 2), primary hyperparathyroidism (*n* = 1), von Willebrand disease (*n* = 1), and osteoarthritis (*n* = 2). Several pertinent parameters on CBC and serum chemistry are summarized in Table 1.

**Table 1:** Pertinent parameters obtained on complete blood count (CBC) and serum chemistry in 53 dogs diagnosed with hypothyroidism

Parameters	Reference interval (*R*.I.)	Median (range)	% Below R.I.	% Above R.I.
**CBC**				
Hematocrit (%)	39–56%	41.9 (28.4–53.3)	32% (17/53)	0% (0/53)
**Serum chemistry**				
Cholesterol (mg/dL or mmol/L)	104–228 mg/dL 2.7–5.9 mmol/L	491 (174-1,829) mg/dL 12.7 (4.5–47.3) mmol/L	0%	91% (48/53)
Alkaline phosphatase (ALP) activity (U/L)	9–90 U/L	85 (12 − 4,934)	0%	45% (24/53)
Alanine transaminase (ALT) activity (U/L)	19–59 U/L	55 (14 − 10,196)	2% (1/53)	45% (24/53)
Creatinine kinase (U/L)	51–418 U/L	215 (54 − 3,952)	0%	11% (6/53)
Urea (mg/dL or mmol/L)	9.8–31.9 mg/dL 3.5–11.4 mmol/L	19.3 (8.1-107.5) mg/dL 6.9 (2.9–38.4) mmol/L	2% (1/53)	19% (10/53)
Creatinine (mg/dL or µmol/L)	0.46–1.37 mg/dL 41–121 µmol/L	1.2 (0.5–3.3) mg/dL 102 (40–292) µmol/L	2% (1/53)	30% (16/53)

**Fig. 1:**
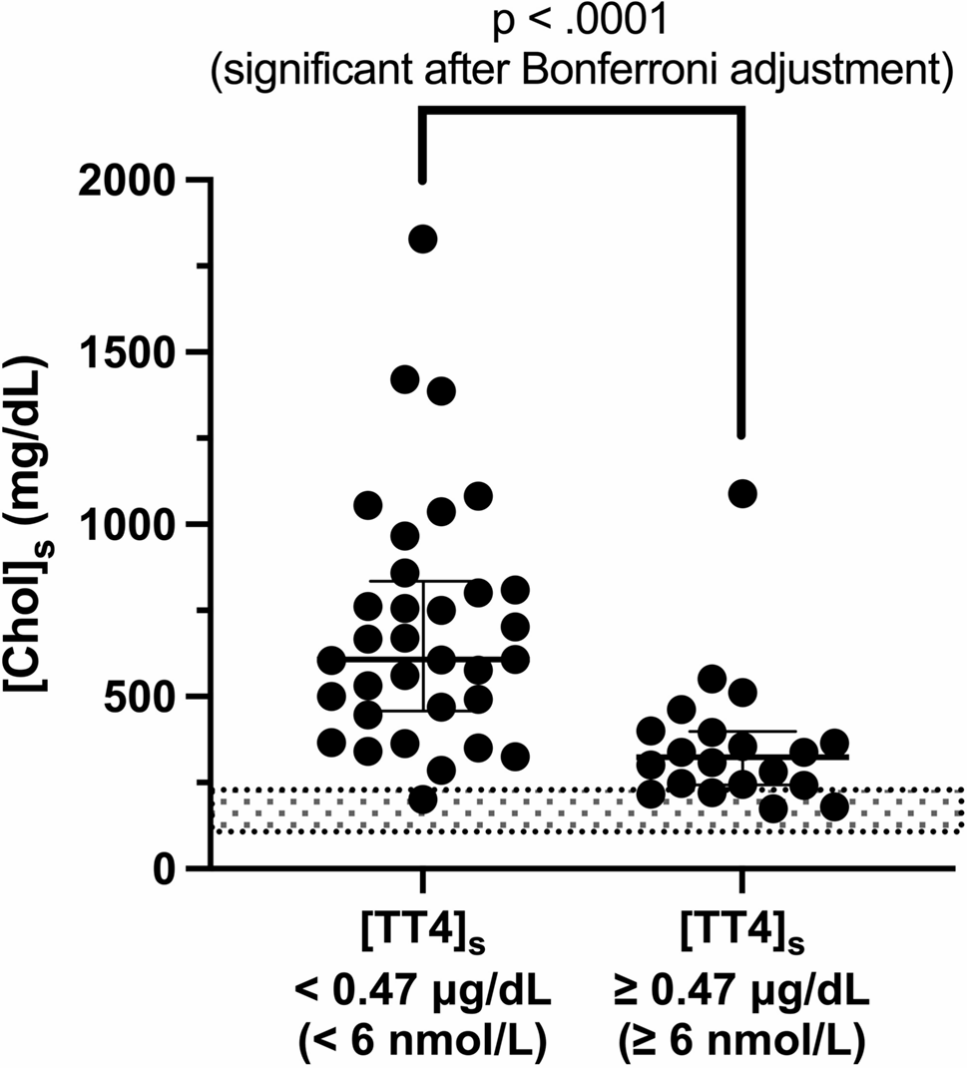
Serum cholesterol concentration ([Chol]_s_) in hypothyroid dogs with serum thyroxine concentration ([TT4]_s_) < 0.47 µg/dL (*n* = 33) or ≥ 0.47 µg/dL (*n* = 20) (i.e. < or ≥ 6 nmol/L). Median values with interquartile ranges are presented (Mann-Whitney Wilcoxon test), and the reference interval for [Chol]_s_ is indicated by the grey area (104–228 mg/dL)

Median [Chol]_s_ for all hypothyroid dogs was 492 mg/dL (12.7 mmol/L) (range: 174–1829 mg/dL or 4.5–47.3 mmol/L), and hypercholesterolemia was reported in 48/53 (91%) of them. Median [Chol]_s_ was significantly higher in dogs with [TT4]_s_ < 0.47 µg/dL (median A = 607 mg/dL or 15.7 mmol/L), than in dogs with [TT4]_s_ ≥ 0.47 µg/dL (median B = 324 mg/dL or 8.4 mmol/L) (median B – median A = -299 mg/dL; 95.21% CI of difference = [-433; -166]; *p* < .0001) (Fig. 1), albeit there was significant overlap between groups.

Serum total thyroxine concentration was below the detection limit (< 0.47 µg/dL or < 6 nmol/L) in 32/53 (60%) dogs. Serum thyroid stimulating hormone concentration ([TSH]_s_) was available in all cases, with a median [TSH]_s_ of 1.44 ng/mL (range 0.05-12.0), and elevated [TSH]_s_ was reported in 47/53 (85%) cases. The [TSH]_s_ of the 6 remaining dogs were within normal reference at 0.05, 0.13, 0.33, 0.33, 0.38, and 0.40 ng/mL (R.I. 0.03–0.58 ng/mL), with [TT4]_s_ of < 0.47, 0.80, 0.83, < 0.47,<0.47 and < 0.47 µg/dL (R.I. 0.93–3.11 µg/dL), respectively. Free T4 ([fT4]_s_) was measured in 8/53 (15%) cases and was decreased in 7/8 (88%) of these cases, including 4 cases with [fT4]_s_ below the limit of detection (< 0.3 ng/dL or < 3.86 pmol/L).

### Diabetes mellitus

Fifty-four dogs had a diagnosis of DM, with a median age of 9 years old (range: 4–15 yo). The most commonly affected breeds were Labrador Retriever (7/54, 13%), Shih Tzu (7/54, 13%) and Bichon Frisé (4/54, 7%), and twenty four other breeds were reported with a prevalence of 2–5%. The DM group included no breeds reported to be predisposed to primary hypercholesterolemia [1]. Sex distribution was almost equal, with 26 (48%) male (*n* = 23 neutered and *n* = 3 intact) and 28 (52%) female (*n* = 21 spayed and *n* = 7 intact) dogs. Nineteen dogs were diagnosed with non-complicated DM and 35 with complicated DM (32 dogs with diabetic ketoacidosis [DKA] and 3 with hyperosmolar hyperglycemic syndrome [HHS]). Other comorbidities were present in 13/54 (24%) cases: chronic kidney disease (*n* = 2), cardiac diseases (degenerative mitral valve disease [*n* = 1], uncharacterized heart murmur [*n* = 1]), urinary diseases (bacterial cystitis [*n* = 1], cystoliths [*n* = 1]), chronic enteropathy (*n* = 1) and mammary gland tumour (*n* = 1). Several pertinent parameters obtained on serum chemistry are summarized in Table 2.

**Table 2:** Pertinent parameters on serum chemistry in 19 dogs with non-complicated diabetes mellitus (DM) and 35 dogs with complicated DM (i.e., diabetic ketoacidosis and hyperosmolar hyperglycemic syndrome). After Bonferroni’s adjustment, a p-value < 0.0083 (< 0.05/6) was considered statistically significant. MW: Mann-Whitney Wilcoxon test. (*) indicates statistically significant results

Serum parameters	Reference interval (*R*.I.)	Median, range	% Below R.I.	% Above R.I.	*p*-value
Cholesterol (mg/dL or mmol/L)	104–228 mg/dL 2.7–5.9 mmol/L	321 (116–928) mg/dL 8.3 (3.0–24.0) mmol/L	0%	85% (46/54)	
Uncomplicated DM		282 (189–928) mg/dL 7.3 (4.9–24.0) mmol/L	0%	80% (15/19)	*p* = .450 (MW)
Complicated DM		322 (116–607) mg/dL 8.34 (3.0-15.7) mmol/L	0%	86% (30/35)	
Blood glucose (mg/dL or mmol/L) – overall	56–114 mg/dL 3.1–6.3 mmol/L	463 (279-1,113) mg/dL 25.7 (15.5–61.8) mmol/L	0%	100% (54/54)	
Uncomplicated DM		418 (279–886) mg/dL 23.2 (15.5–49.2) mmol/L	0%	100% (19/19)	*p* = .130 (MW)
Complicated DM		483 (317-1,113) mg/dL 27.6 (17.6–61.8) mmol/L	0%	100% (35/35)	
Alkaline phosphatase (ALP) (U/L) – overall	9–90 U/L	515 (23 − 4,216)	0%	96% (52/54)	
Uncomplicated DM		233 (23 − 1,349)	0%	17/19 (89%)	*p* = .019 (MW)
Complicated DM		610 (99 − 4,216)	0%	35/35 (100%)	
Alanine transaminase (ALT) (U/L) – overall	19–59 U/L	119 (22–447)	0%	78% (42/54)	
Uncomplicated DM		103 (32–329)	0%	68% (13/19)	*p* = .205 (MW)
Complicated DM		123 (22–447)	0%	83% (29/35)	
Urea (mg/dL or mmol/L) – overall	9.8–31.9 mg/dL 3.5–11.4 mmol/L	24.4 (7.8-167.2) mg/dL 8.7 (2.8–59.7) mmol/L	4% (2/54)	37% (20/54)	
Uncomplicated DM		19.3 (9.2–61.0) mg/dL 6.9 (3.3–21.8) mmol/L	5% (1/19)	21% (4/19)	*p* = .064 (MW)
Complicated DM		28.6 (7.8–167) mg/dL 10.2 (2.8–59.7) mmol/L	3% (1/35)	46% (16/35)	
Creatinine (mg/dL or µmol/L) – overall	0.46–1.37 mg/dL 41–121 µmol/L	0.9 (0.4–5.2) mg/dL 77 (39–455) µmol/L	6% (3/54)	17% (9/54)	
Uncomplicated DM		0.9 (0.4–2.3) mg/dL 80 (39–206) µmol/L	11% (2/19)	5% (1/19)	*p* = .352 (MW)
Complicated DM		0.9 (0.4–5.2) mg/dL 76 (39–455) µmol/L	3% (1/35)	23% (8/35)	

**Fig. 2:**
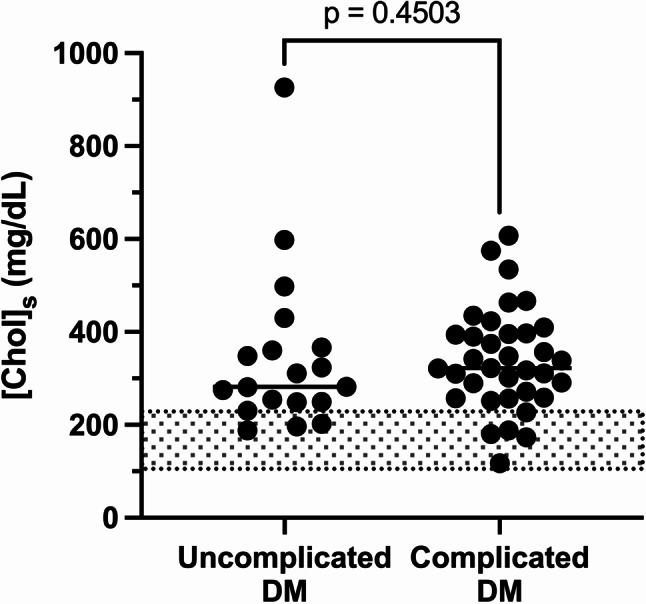
Serum cholesterol concentration ([Chol]_s_) in dogs with uncomplicated diabetes mellitus (DM) (*n* = 19) and complicated DM (*n* = 35). Median values with interquartile ranges are presented (Mann-Whitney Wilcoxon test), and the reference interval for [Chol]_s_ is indicated by the grey area (104–228 mg/dL)

When both complicated and uncomplicated DM groups were combined, median [Chol]_s_ was 321 mg/dL (8.3 mmol/L) (range: 116–928 mg/dL or 3.0–24.0 mmol/L), and hypercholesterolemia was present in 46/54 (85%) dogs. In dogs with uncomplicated DM, and the two forms of complicated DM (DKA and HHS), median [Chol]_s_ was 282 mg/dL (7.3 mmol/L) (range: 189–928 mg/dL or 4.9–24.0 mmol/L), 333 mg/dL (8.6 mmol/L) (range: 116–607 mg/dL or 3.0-15.7 mmol/L), and 290 mg/dL (7.5 mmol/L) (range: 174–468 mg/dL or 4.5–12.1 mmol/L), respectively (*p* = .519). No significant difference was found between [Chol]_s_ in dogs with uncomplicated DM (median A) *versus* complicated DM (median B) (median B - median A = + 28 mg/dL; 95.02% CI of difference = [-38; +83]; *p* = .450) (Fig. 2). In dogs with DKA, no significant difference in mean [Chol]_s_ was found without (*n* = 27; mean A = 345 mg/dL or 8.8 mmol/L, range: 116–607 mg/dL or 3.0-15.7 mmol/L) or with (*n* = 5; mean B = 326 mg/dL or 8.2 mmol/L, range 271–410 mg/dL or 7.0-10.6 mmol/L) concurrent acute pancreatitis (mean B - mean A = -19 mg/dL; 95% CI of difference = [-129; +90]; *p* = .720). No significant difference was found between median [Chol]_s_ in DM dogs with a serum blood glucose < 450 mg/dL or < 25 mmol/L (*n* = 26, median A = 317 mg/dL or 8.2 mmol/L, range: 182–928 mg/dL or 4.7–24.0 mmol/L) *versus* ≥ 450 mg/dL or ≥ 25 mmol/L (*n* = 28, median B = 320 mg/dL or 8.3 mmol/L, range 116–607 mg/dL or 3.0-15.7 mmol/L) (median B - median A = + 17 mg/dL; 95% CI of difference = [-51; +73]; *p* = .612).

### Hyperadrenocorticism

Sixty-two dogs had a diagnosis of HAC, with a median age of 10 years old (range: 5–16 yo). The most common breed affected was the Shih Tzu (13/62, 21%), followed by Bichon Frisé (6/62, 10%), and thirty-one other breeds were reported with a prevalence of 2–5%. The HAC group included the following breeds reported to be predisposed to primary hypercholesterolemia: Miniature Schnauzer (*n* = 3) and Beagle (*n* = 1) [1]. Sex distribution was almost equal, with 28/62 (45%) male (*n* = 26 neutered and *n* = 2 intact) and 34/62 (55%) female (*n* = 33 spayed and *n* = 1 intact) dogs. Pituitary-dependent hyperadrenocorticism (PDH) was diagnosed in 48/62 (77%) dogs, and adrenal-dependent hyperadrenocorticism (ADH) in 6/62 (10%). The underlying cause for HAC (PDH *versus* ADH) was not reported in the remaining 8 dogs. A low-dose dexamethasone suppression test (LDDST) test was performed in 56/62 (90%) dogs, and an ACTH stimulation test (ACTHST) was performed in 12/62 (19%) dogs, of which 6 dogs did not have an LDDST performed. A high-dose dexamethasone suppression test (HDDST) was performed in only 3 dogs, and a urine cortisol: creatinine ratio (UCCR) was measured in a single dog. Comorbidities included cardiac diseases (degenerative mitral valve disease [*n* = 2], pulmonary hypertension [*n* = 1]), neurological disease (idiopathic epilepsy [*n* = 1]), urinary diseases (protein-losing nephropathy [*n* = 1], nephroliths/uroliths [*n* = 1], chronic kidney disease [*n* = 1]), respiratory disease (tracheal collapse [*n* = 2]), dermatologic disease (pemphigus foliaceus [*n* = 1]), chronic enteropathy (*n* = 1), soft tissue sarcoma (*n* = 1), and acute pancreatitis (*n* = 1 – for whom [Chol]_s_ was 7.9 mmol/L). Several pertinent parameters on serum chemistry are summarized in Table 3. Ultrasonographic measurements of the adrenal glands were available in 45/62 (73%) dogs. The median width of the left caudal pole was 8 mm (range: 3–35 mm), and the median width of the right caudal pole was 8.5 mm (range: 5–41 mm). The remaining 17/62 (27%) dogs did not have abdominal ultrasound performed. Only 4/46 (8%) dogs for whom an abdominal ultrasound was performed had sonographic features of semi-organized gall bladder debris ([Chol]_s_ = 212, 314, 339, and 479 mg/dL), but none had evidence of extrahepatic biliary tract obstruction (EHBO).

**Table 3:** Pertinent parameters from the serum biochemistry profile in 62 dogs diagnosed with hyperadrenocorticism. MW: Mann-Whitney Wilcoxon test

Serum parameters	Reference interval (*R*.I.)	Median, range	% Below R.I.	% Above R.I.
Cholesterol (mg/dL or mmol/L)	104–228 mg/dL 2.7–5.9 mmol/L	309 (151–630) mg/dL 8.0 (3.9–16.3) mmol/L	0%	81% (50/62)
Alkaline phosphatase (ALP) (U/L)	9–90 U/L	540 (25-5471)	0%	92% (57/62)
Alanine transaminase (ALT) (U/L)	19–59 U/L	125 (4-2187)	2% (1/62)	73% (45/62)
Urea (mg/dL or mmol/L)	9.8–31.9 mg/dL 3.5–11.4 mmol/L	17.5 (7.3–48.7) mg/dL 6.25 (2.6–17.4) mmol/L	3% (2/62)	8% (5/62)
Creatinine (mg/dL or µmol/L)	0.46–1.37 mg/dL 41–121 µmol/L	0.7 (0.2–1.5) mg/dL 61 (17–130) µmol/L	11% (7/62)	2% (1/62)
Lipase (U/L)	0-701 U/L	153 (34-3281)	0%	18% (10/56)

**Fig. 3:**
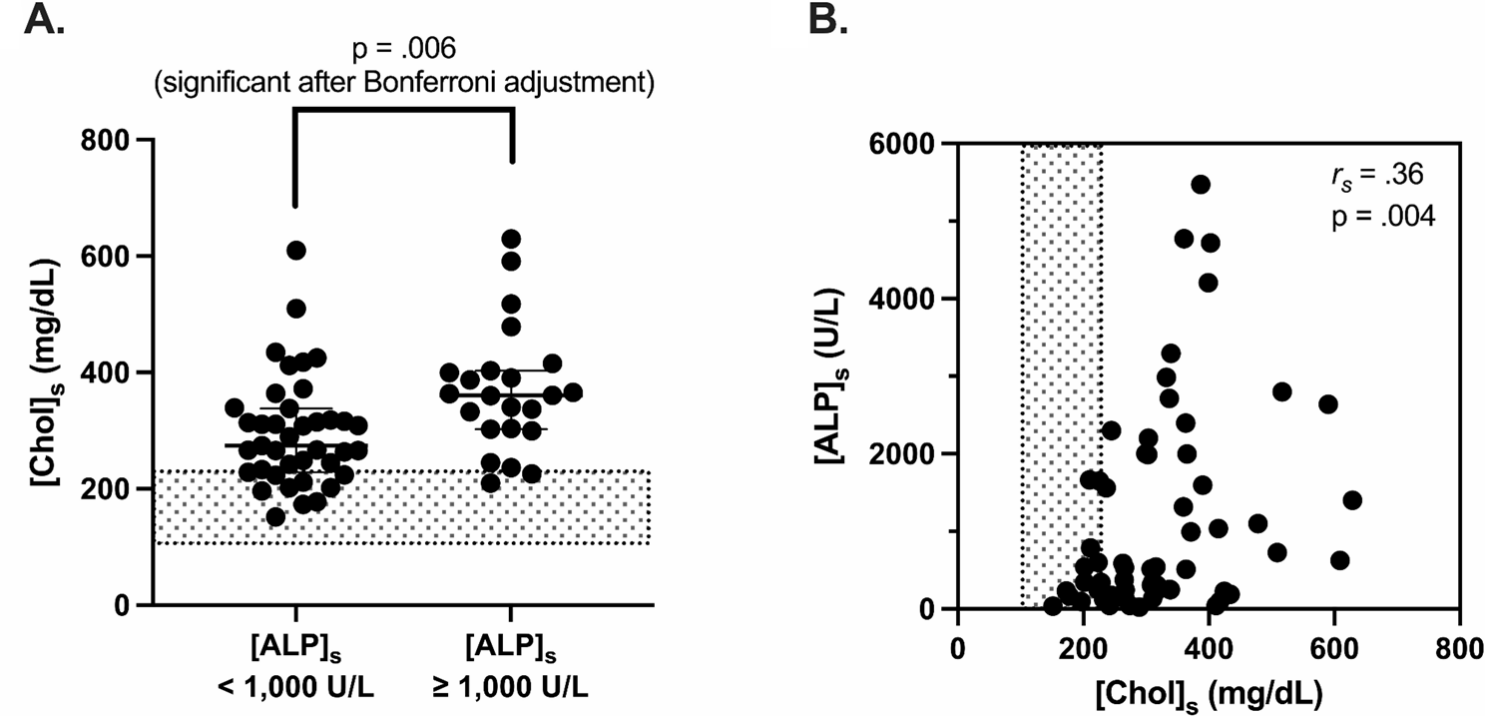
(**A**) Serum cholesterol concentration ([Chol]_s_) in dogs with hyperadrenocorticism and serum alkaline phosphate activity ([ALP]_s_) < 1,000 (*n* = 39) or ≥ 1,000 U/L (*n* = 23). Median values with interquartile ranges are presented (Mann-Whitney Wilcoxon test). (**B**) Correlation between [Chol]_s_ and [ALP]_s_ in 62 dogs with hyperadrenocorticism. *r*_*s*_:Spearmann correlation coefficient. The reference interval for [Chol]_s_ is indicated by the grey areas (104–228 mg/dL)

Overall, median [Chol]_s_ was 309 mg/dL or 8.0 mmol/L (range: 151–630 mg/dL or 3.9–16.3 mmol/L), with hypercholesterolemia present in 50/62 (81%) HAC dogs. Median [Chol]_s_ was significantly lower in dogs with HAC that had [ALP]_s_ < 1,000 U/L (median A = 275 mg/dL or 7.1 mmol/L, range 151–611 mg/dL or 3.9–15.8 mmol/L) *versus* ≥ 1,000 U/L (median B = 360 mg/dL or 9.3 mmol/L, range 209–630 mg/dL or 5.4–16.3 mmol/L) (median B - median A = + 74 mg/dL; 95.14% CI of difference = [+ 25; +121]; *p* = .006) (Fig. 3A). There was a positive and moderate correlation between [Chol]_s_ and [ALP]_s_ (*r*_*s*_ = 0.36, *p* = .004) (Fig. 3B).

### Hypoadrenocorticism

Seventy-nine dogs were diagnosed with HA, with a median age of 5 years old (range: 1.5 mo – 15 yo). The most common breed affected was the Standard Poodle (7/79, 9%), followed by Labradoodle (6/79, 8%) and Shih Tzu 6/79 (8%), Labrador Retriever (5/79, 6%) and Maltese (4/79, 5%), and thirty four other breeds were reported with a prevalence of 1–4%. The HA group included the following breeds reported to be predisposed to primary hypercholesterolemia: Miniature Schnauzer (*n* = 3) and Beagle (*n* = 1) [1]. Sex distribution was almost equal, with 38/79 (48%) male (*n* = 32 neutered and *n* = 6 intact) and 41/79 (52%) female (*n* = 35 spayed and *n* = 6 intact) dogs. No pertinent comorbidities were reported. An ACTHST was performed in all cases; 76/79 (96%) dogs had a pre-ACTH serum cortisol concentration < 0.73 µg/dL (< 20 nmol/L) with the remaining 3 cases having a mean pre-ACTH serum cortisol concentration of 1.34 µg/dL (37 nmol/L) (range: 1.12–1.70 µg/dL or 31–47 nmol/L), and 74/79 (94%) dogs had a post-ACTH serum cortisol concentration < 0.73 µg/dL (< 20 nmol/L) with the remaining 5 cases having a mean post-ACTH serum cortisol concentration of 1.27 µg/dL (35 nmol/L) (range: 0.83–1.67 µg/dL or 23–46 nmol/L). Plasma endogenous ACTH concentration ([eACTH]_p_) was measured in 5/79 (6%) dogs: of these, 1 dog was diagnosed with secondary hypoadrenocorticism ([eACTH]_p_ = 22.3 pg/mL or 4.9 pmol/L), and 4 dogs were diagnosed with primary hypoadrenocorticism (mean [eACTH]_p_ = 786 pg/mL or 173 pmol/L, range 577–904 pg/mL or 127–199 pmol/L). Overall, the median serum sodium: potassium concentration ratio (Na/K ratio) was 21 (range 11.8–45). Fifty-five dogs were diagnosed with both hyponatremic and/or hyperkalemic hypoadrenocorticism (HHHA) based on a consistent ACTHST and documentation of a low Na/K ratio, and 24 dogs were initially diagnosed with eunatremic eukalemic hypoadrenocorticism (EEHA) based on a consistent ACTHST and absence of significant electrolyte abnormalities at the time of diagnosis. In dogs diagnosed with HHHA, the median Na/K ratio was 20 (range 19.6–27), whereas in dogs diagnosed with EEHA, the median Na/K ratio was 31 (range 27–45). Other pertinent parameters obtained on serum chemistry are summarized in Table 4.

**Table 4:** Pertinent parameters on serum chemistry in 79 dogs diagnosed with hypoadrenocorticism, including 55 dogs with hyponatremic and/or hyperkalemic hypoadrenocorticism (HHHA) and 24 dogs with eunatremic eukalemic hypoadrenocorticism (EEHA). After Bonferroni’s adjustment, a *p*-value < 0.0055 (< 0.05/9) was considered statistically significant. MW: Mann-Whitney Wilcoxon test. (*) indicates statistically significant results

Serum parameters	Reference interval (*R*.I.)	Median, range	% Below R.I.	% Above R.I.	*p*-value
Cholesterol (mg/dL or mmol/L) - overall	104–228 mg/dL 2.7–5.9 mmol/L	112 (31–309) mg/dL 2.9 (0.8-8.0) mmol/L	43% (34/79)	9% (7/79)	
HHHA		124 (53–309) mg/dL 3.2 (1.36-8.0) mmol/L	36% (20/55)	13% (7/55)	*p* = .033 (MW)
EEHA		104 (31–205) mg/dL 2.7 (0.8–5.3) mmol/L	58% (14/24)	0%	
Alkaline phosphatase (ALP) (U/L) – overall	9–90 U/L	35 (8-946)	3% (2/79)	10% (8/79)	
HHHA		35 (8-946)	2% (1/55)	7% (4/55)	*p* = .190 (MW)
EEHA		46 (5-825)	4% (1/24)	17% (4/24)	
Alanine transaminase (ALT) (U/L) – overall	19–59 U/L	55 (4-519)	3% (2/79)	43% (34/79)	
HHHA		56 (4-271)	4% (2/55)	44% (24/55)	*p* = .453 (MW)
EEHA		54.5 (33–519)	0%	42% (10/24)	
Blood glucose (BG) (mg/dL or mmol/L) – overall	56–114 mg/dL 3.1–6.3 mmol/L	83 (20–353) mg/dL 4.6 (1.1–19.6) mmol/L	19% (15/79)	15% (12/79)	
HHHA		86 (20–353) mg/dL 4.8 (1.1–19.6) mmol/L	15% (8/55)	20% (11/55)	*p* = .066 (MW)
EEHA		76 (31–133) mg/dL 4.2 (1.7–7.4) mmol/L	29% (7/24)	4% (1/24)	
Urea (BUN) (mg/dL or mmol/L) – overall	9.8–31.9 mg/dL 3.5–11.4 mmol/L	44.8 (5.9-126.6) mg/dL 16 (2.1–45.2) mmol/L	4% (3/79)	59% (47/79)	
HHHA		57.7 (6.7-118.7) mg/dL 20.6 (2.4–42.4) mmol/L	2% (1/55)	82% (45/55)	*p* < .0001* (MW)
EEHA		16.8 (5.9-126.6) mg/dL 6.0 (2.1–45.2) mmol/L	8% (2/24)	8% (2/24)	
Creatinine (mg/dL or µmol/L) – overall	0.46–1.37 mg/dL 41–121 µmol/L	1.4 (0.4–5.3) mg/dL 123 (33–468) µmol/L	1% (1/79)	53% (42/79)	
HHHA		1.6 (0.6–5.3) mg/dL 144 (49–468) µmol/L	0%	69% (38/55)	*p* < .0001* (MW)
EEHA		0.9 (0.4–1.8) mg/dL 78.5 (33–160) µmol/L	4% (1/24)	17% (4/24)	
Total proteins (TP) – overall	55–71 g/L	61 (30–89)	30% (24/79)	11% (9/79)	
HHHA		61 (30–89)	29% (16/55)	15% (8/55)	*p* = .265 Unpaired t-test
EEHA		59.5 (34–85)	33% (8/24)	4% (1/24)	
Albumin (g/L) – overall	28–38 g/L	30 (15–43)	34% (27/79)	13% (10/79)	
HHHA		32 (15–43)	22% (12/55)	18% (10/55)	*p* = .0005* (MW)
EEHA		24.5 (15–36)	63% (15/24)	0%	
Total calcium (tCa) (mg/dL or mmol/L)	7.7–12.1 mg/dL 1.91–3.03 mmol/L	10.5 (4.5–17.0) mg/dL 2.61 (1.61–4.25) mmol/L	5% (4/79)	24% (19/79)	
HHHA		11.4 (4.5–17.0) mg/dL 2.84 (1.61–4.25) mmol/L	4% (2/55)	35% (19/55)	*p* < .0001* (MW)
EEHA		9.5 (6.9–11.1) mg/dL 2.37 (1.73–2.77) mmol/L	8% (2/24)	0%	

**Fig. 4:**
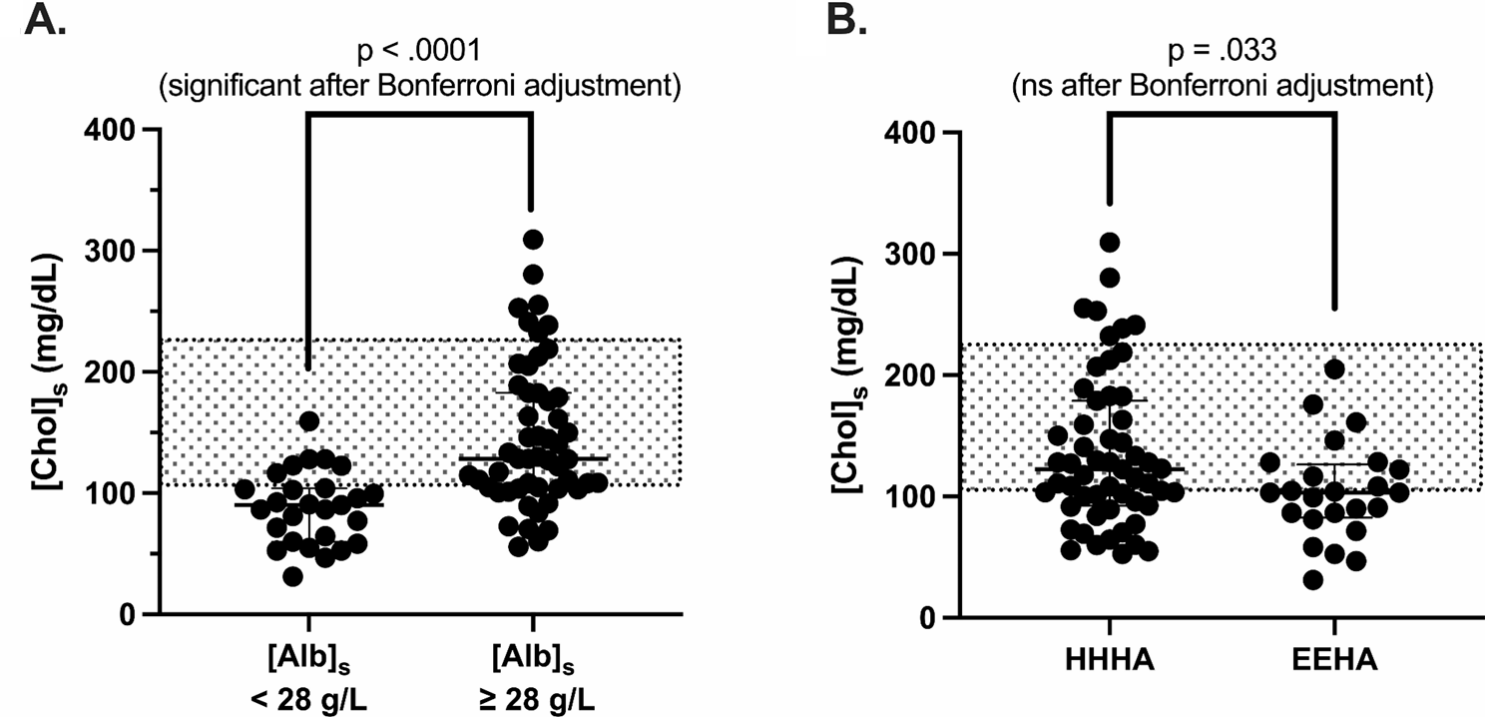
(**A**) Serum cholesterol concentration ([Chol]_s_) in dogs with hypoadrenocorticism (HA) and serum albumin concentration ([Alb]_s_) < 28 g/L (*n* = 27) and ≥ 28 g/L (*n* = 52). (**B**) – Serum [Chol]_s_ in dogs with eunatremic eukalemic hypoadrenocorticism (EEHA) (*n* = 24), and in dogs with hyponatremic and/or hyperkalemic hypoadrenocorticism (HHHA) (*n* = 55). Median values with interquartile ranges are presented (Mann-Whitney Wilcoxon test), and the reference interval for [Chol]_s_ is indicated by the grey areas (104–228 mg/dL)

In all dogs with HA, median [Chol]_s_ was 112 mg/dL (or 2.9 mmol/L) (range: 31–309 mg/dL or 0.8-8.0 mmol/L), with hypocholesterolemia, normocholesterolemia and hypercholesterolemia present in 34/79 (43%), 38/79 (48%) and 7/79 (9%) dogs, respectively. Median [Chol]_s_ was significantly lower in dogs with [Alb]_s_ < 28 g/L (median A = 89 mg/dL or 2.3 mmol/L) vs. ≥ 28 g/L (median B = 128 mg/dL or 3.3 mmol/L) (median B - median A = + 49 mg/dL; 95.03% CI of difference = [+ 27; +72]; *p* < .0001) (Fig. 4A). No significant difference was found between median [Chol]_s_ in dogs with HHHA (median A = 124 mg/dL or 3.2 mmol/L) and in dogs with EEHA (median B = 104 mg/dL or 2.7 mmol/L) (median B - median A = -22 mg/dL; 95.00% CI of difference = [-47; -1]; *p* = .033) after Bonferroni’s adjustment (Fig. 4B).

### Linear regression results

When [Chol]_s_ was used as a predictor variable, the outcome variables [TT4]_s_, [TSH]_s_, serum blood glucose concentration, [ALP]_s_, post-ACTHST serum cortisol concentration, and serum cortisol concentration at T8h after LDDST, did not meet the requirement for linear regression.

**Fig. 5:**
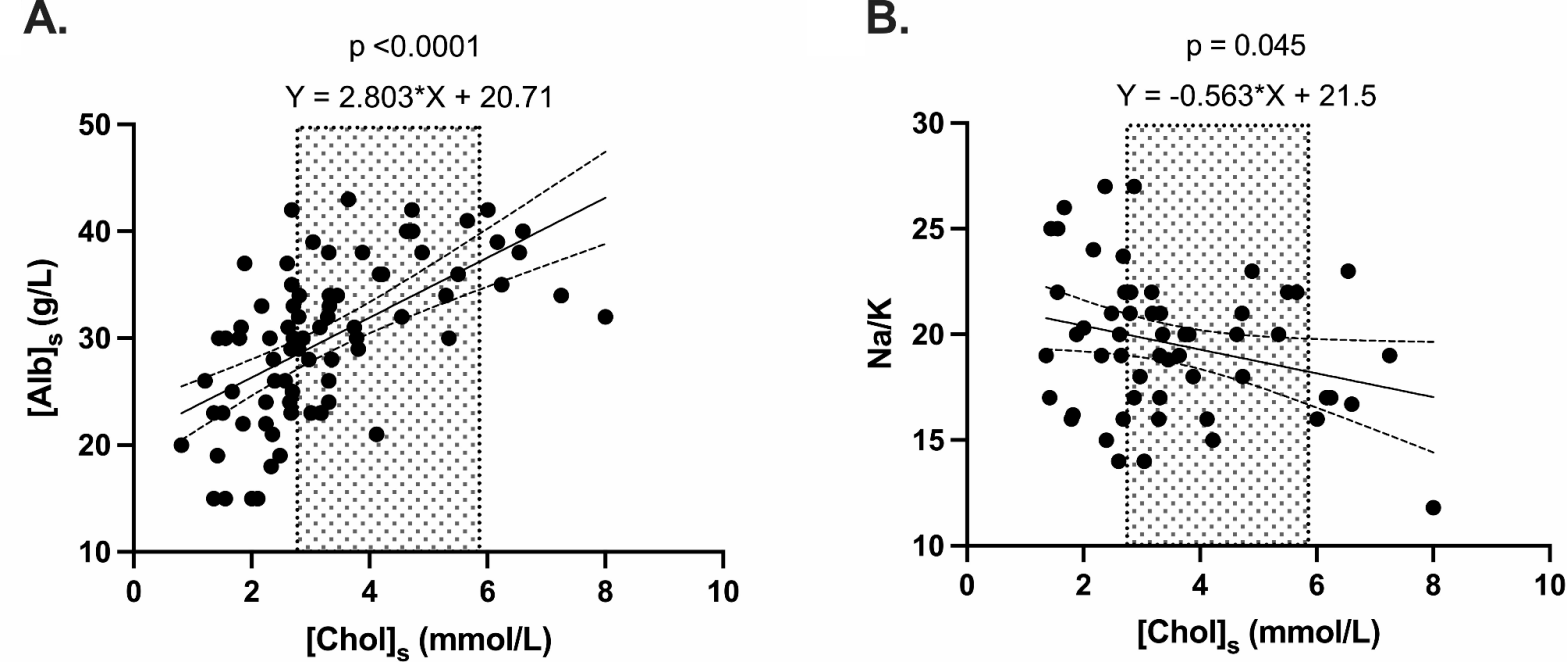
(**A**) Association between serum cholesterol concentration ([Chol]_s_) (predictor) and serum albumin concentration ([Alb]_s_) (outcome) in dogs with hypoadrenocorticism (HA) (linear regression). (**B**) Association between serum [Chol]_s_ (predictor) and serum sodium: potassium concentration (Na/K) (outcome) in dogs with hyponatremic and/or hyperkalemic hypoadrenocorticism (linear regression). The reference interval for [Chol]_s_ is indicated by the grey areas (2.7–5.9 mmol/L)

When [Chol]_s_ was used as a predictor variable, the outcome variable [Alb]_s_ met the requirements for linear regression when including all HA dogs. For every decrease of 1 mmol/L (or 39 mg/dL) of [Chol]_s_, [Alb]_s_ decreased by 2.8 g/L (95% C.I. = [1.93; 3.67]), and for every decrease of 1 g/L of [Alb]_s_, [Chol]_s_ decreased by 0.12 mmol/L (or 5 mg/dL) (95% C.I. = [0.08; 0.16]) (*p* < .0001, linear regression) (Fig. 5A).

When [Chol]_s_ was used as a predictor variable, the outcome variable Na/K met the requirements for linear regression in dogs with HHHA. In HHHA dogs, for every increase in 1 mmol/L (or 39 mg/dL) of [Chol]_s_, the Na/K ratio decreased by 0.56 (95% C.I. = [-1.11; -0.01]), and for every decrease of 1 unit of the serum Na/K ratio, [Chol]_s_ increased by 0.13 mmol/L (or 5 mg/dL) (95% C.I. = [-0.26; -0.003]) (*p* = .045, linear regression) (Fig. 5B).

### All endocrinopathies

**Fig. 6:**
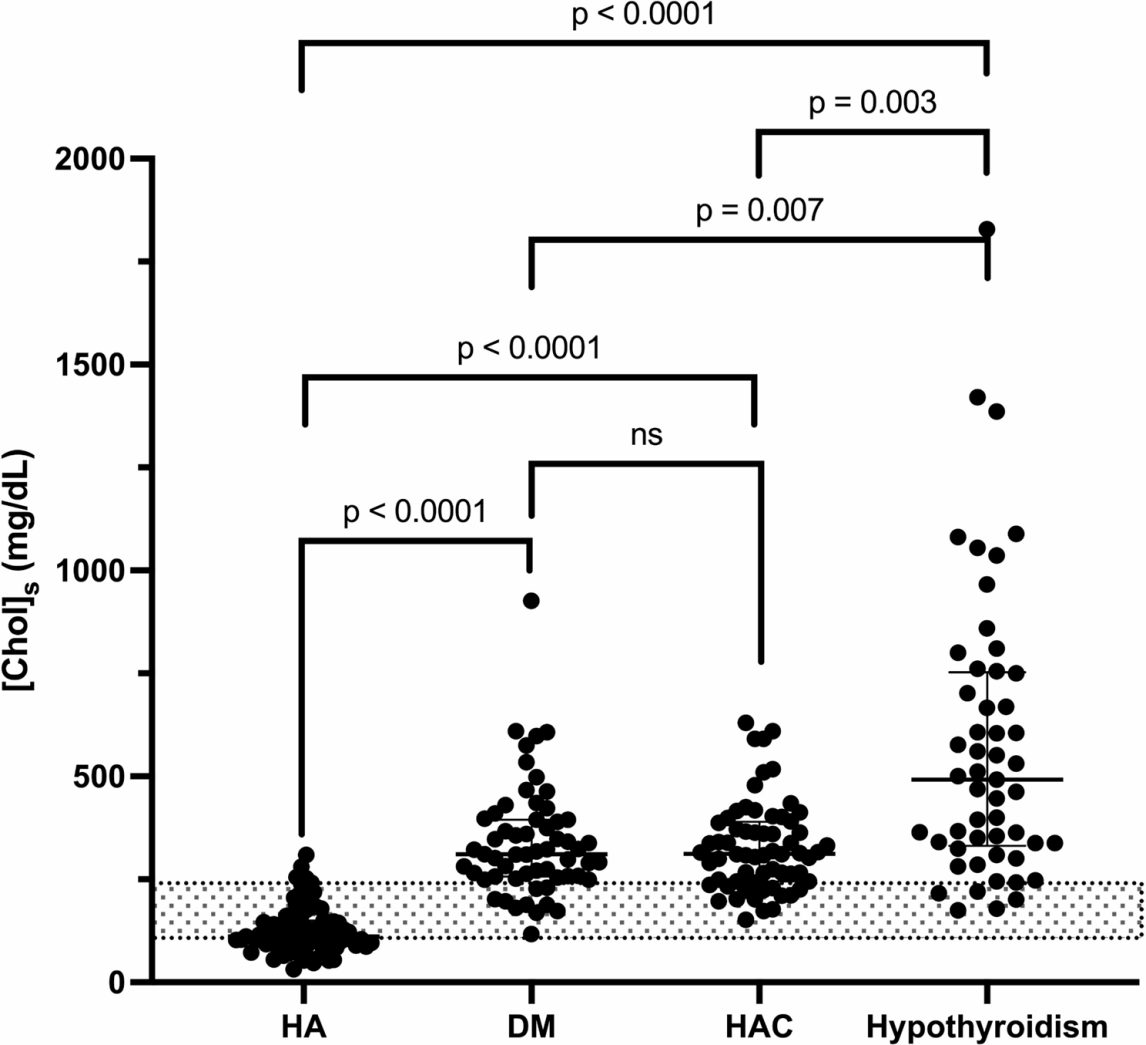
Serum cholesterol concentration ([Chol]_s_) in dogs with hypothyroidism (*n* = 53), diabetes mellitus (DM) (*n* = 54), hyperadrenocorticism (HAC) (*n* = 62), and hypoadrenocorticism (HA) (*n* = 79), compared using a Dunn’s pairwise comparison with Bonferroni correction. Median values with interquartile ranges are presented, and the reference interval for [Chol]_s_ is indicated by the grey area (104–228 mg/dL)

Table 5; Fig. 6 summarize the median [Chol]_s_ for each endocrinopathy in dogs described previously (hypothyroidism, DM, HAC and HA).

**Table 5:** Correlation matrix of the median serum cholesterol concentration ([Chol]_s_) for dogs diagnosed with each of the following endocrinopathies - hypothyroidism (HypoT, *n* = 53), diabetes mellitus (DM, *n* = 54), hyperadrenocorticism (HAC, *n* = 62), and hypoadrenocorticism (HA, *n* = 79). Comparison was performed using Dunn’s pairwise comparison with Bonferroni correction test. (*) indicates statistically significant results

Correlation matrix of median [Chol]_s_	HA	DM	HAC
DM	112 (HA) vs. 321 (DM) mg/dL 2.9 (HA) vs. 8.3 (DM) mmol/L *p* < .0001*		
HAC	112 (HA) vs. 309 (HAC) mg/dL 2.9 (HA) vs. 8.0 (HAC) mmol/L *p* < .0001*	321 (DM) vs. 309 (HAC) mg/dL 8.3 (DM) vs. 8.0 (HAC) mmol/L *p* = 1	
HypoT	112 (HA) vs. 491 (hypoT) mg/dL 2.9 (HA) vs. 12.7 (hypoT) mmol/L *p* < .0001*	321 (DM) vs. 491 (hypoT) mg/dL 8.3 (DM) vs. 12.7 (hypoT) mmol/L *p* = .007*	309 (HAC) vs. 491 (hypoT) mg/dL 8.0 (HAC) vs. 12.7 (hypoT) mmol/L *p* = .003*

## Discussion

Our study aimed to investigate serum cholesterol disturbances at the time of diagnosis in dogs with various common endocrinopathies. We identified that hypercholesterolemia was most commonly observed in hypothyroidism (91%), followed by DM (85%) and HAC (81%) at the time of diagnosis, albeit there were no significant differences between the two latter groups. We also evaluated the association between [Chol]_s_ and changes in common laboratory measurements.

**Fig. 7:**
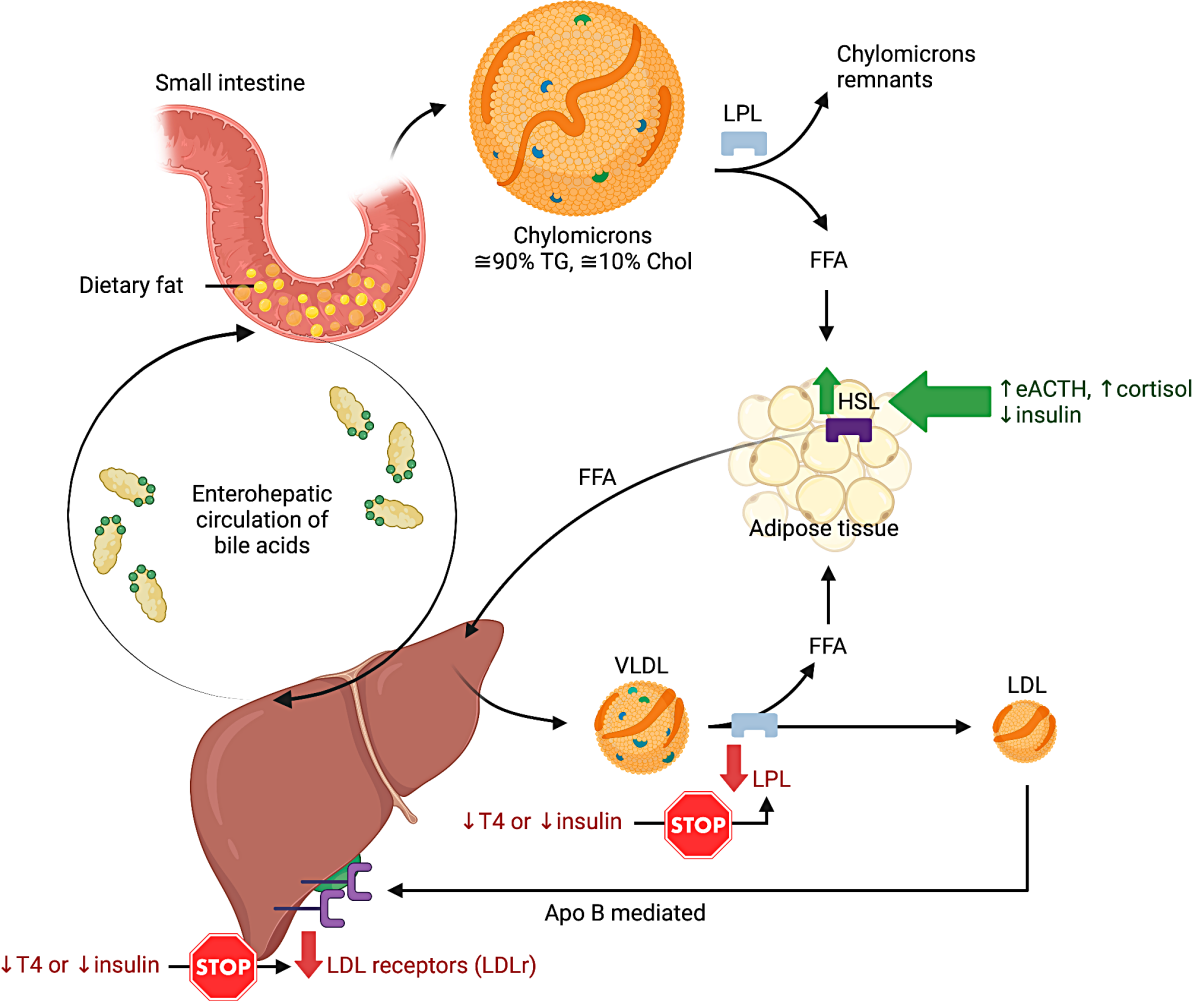
Influence of thyroxin, insulin, cortisol and endogeneous adrenocorticotropic hormone on the major pathways of lipid metabolism. eACTH: endogeneous adrenocorticotropic hormone; Chol: cholesterol; FFA: free fatty acid; HSL: hormone-sensitive lipase; LDL: low-density lipoprotein; LDLr: low-density lipoprotein receptor; LPL: lipoprotein lipase; T4:thyroxine; TG: triglycerides; VLDL: very-low density lipoprotein. Adapted from Guyton and Hall (2021) [47]. Created with BioRender.com (Paulin, 2024)

Hypercholesterolemia was observed in 91% of our hypothyroid dogs, similarly to a recent study which reported hypercholesterolemia in 22/24 (91%) hypothyroid dogs [19]. However, this is higher than the prevalence reported in older papers (Panciera et al.., 1994–48/66 [73%]; Dixon et al.., 1999, 38/49 [78%]) [4, 5], or in another recent study (31/38 [81%]) [20]. In our study, median [Chol]_s_ in hypothyroid dogs was 492 mg/dL (range: 174–1829) which is slightly lower than the median [Chol]_s_ of 554 mg/dL (range: 191–1300) reported by Di Paola et al.. (2020) [19], and of 566 mg/dL (range: 206–2025) reported by Corsini et al.. (2021) [20]. Thyroid hormones stimulate nearly all aspects of lipid metabolism, including synthesis, mobilization, and degradation. Both lipid synthesis and degradation are suppressed in hypothyroidism, with degradation being more affected than synthesis [21, 22]. This results in the accumulation of plasma lipids in hypothyroidism and the potential for development of atherosclerosis [22]. The deficiency of thyroid hormones leads to a decrease in hepatic LDL receptor activity and reduced activity of lipoprotein lipase (LPL) and hepatic lipase, which have been proposed as the underlying mechanisms responsible for the lipoprotein cholesterol abnormalities identified in hypothyroid dogs (Fig. 7) [23, 24]. In humans, the higher the original [TSH]_s_ and the elevation of serum LDL, the greater the magnitude of reduction in LDL cholesterol after thyroxine supplementation [25]. However, due to the retrospective nature of the study, the majority of dogs with hypothyroidism had different recheck timelines, some were lost to follow-up after diagnosis, and complete lipoprotein profile is not routinely performed in our institution or in veterinary medicine as a rule; therefore, we were unable to comment on this phenomenon in dogs.

Our study reported a median [Chol]_s_ of 321 mg/dL (range: 116–928) in dogs with DM, which was similar to the median of 369 mg/dL (range: 160-2,067) reported by a previous study which included 37 dogs with uncomplicated DM, 6 with DKA and 5 with ketosis without acidosis [9]. Insulin is a potent inhibitor of lipolysis and free fatty acid (FFA) oxidation. Insulin deficiency leads to decreased LPL activity and increased hormone-sensitive lipase (HSL) activity (Fig. 7). This results in increased hepatic production of triglyceride (TG)-rich very-low-density lipoprotein (VLDL) particles and decreased clearance of VLDL particles [26–29]. The activation of HSL leads to the release of substantial amounts of FFA from adipocytes into the bloodstream. The liver subsequently converts these FFAs into TGs, which are then packaged into VLDL particles and secreted back into the circulation. The increased concentration of intrahepatic cholesterol down-regulates the LDL receptor, thereby reducing the clearance of circulating cholesterol-containing LDL and HDL particles, ultimately leading to hypercholesterolemia [30]. The combination of elevated LDL and reduced HDL cholesterol concentrations may contribute to the accelerated development of atherosclerotic vascular disease and coronary heart disease, which represents the major long-term complication of diabetes in humans [29]. While similar vascular complications have only rarely been documented in diabetic dogs [22], this is presumably due to the predominance of HDLs in dogs (as opposed to LDLs in humans) and the relatively shorter lifespan of dogs, which may limit the development of atherosclerosis [31]. Poorly controlled insulin-dependant DM (type 1) is associated with hypertriglyceridemia, increased serum long-chain FFA concentrations, and hypercholesterolemia in dogs [8, 9]. Insulin stimulates the production of the LDL receptors, thus, hypercholesterolemia appears to be primarily the result of a decrease in receptor-mediated uptake of LDL. Increased intestinal synthesis of cholesterol also appears to play a role in the genesis of hypercholesterolemia in dogs with DM. In our study, median [Chol]_s_ was similar in dogs with uncomplicated DM or DKA, and also similar in dogs with DKA with or without concurrent acute pancreatitis, when diagnosed based on clinical signs, sonographic findings +/- cPLI. Recently, Xenoulis et al.. (2020) reported that most dogs with naturally occurring pancreatitis (> 70%) had serum triglyceride and cholesterol concentrations within reference intervals [32], which supports our findings when excluding dogs with EHBO.

Hypercholesterolemia was observed in 81% of dogs with HAC, which is higher than the prevalence of 66% reported by a recent study [33]. In another retrospective study, hypercholesterolemia was reported in 40%, 63% and 93% of dogs with PDH with no evidence of biliary stasis, *versus* with evidence of cholestasis, or a gallbladder mucocele appreciated on abdominal ultrasound, respectively [34]. Our study reported a median [Chol]_s_ of 309 mg/dL (range: 151–630) in HAC dogs, without distinguishing cases with or without concurrent cholestastic disease using hepatobiliary ultrasound. In a recent study, medians [Chol]_s_ were 228 mg/dL (range: 214–228) and 300 mg/dL (range: 127–1479) in HAC dogs without and with cholestatic disease based on hepatobiliary ultrasound, respectively [35]. In one study abstract, approximately 90% of dogs with HAC had hypercholesterolemia, with approximately 10% of dogs exhibiting [Chol]_s_ <250 mg/dL (< 6.48 mmol/L), 15% between 250 and 300 mg/dL (6.5–7.8 mmol/L), and 75% >300 mg/dL (7.8 mmol/L) [36]. Applying the same [Chol]_s_ intervals to our study, 28%, 12%, and 60% of HAC dogs had [Chol]_s_ <250 mg/dL, between 250 and 300 mg/dL, or > 300 mg/dL, respectively. Our study reported significantly higher [Chol]_s_ in HAC dogs with [ALP]_s_ ≥ 1,000 U/L *versus* < 1,000 U/L, and a positive and moderate correlation between [Chol]_s_ and [ALP]_s_. Higher ALP activity may suggest higher circulating cortisol and subsequently more severe glycogen-associated vacuolar (or steroid-induced) hepatopathy. However, intrahepatic/extrahepatic cholestasis could also be a contributing factor, as 4/46 (8%) HAC dogs displayed sonographic features of semi-organized gallbladder debris, although none had evidence of EHBO. The remaining 17/62 (27%) dogs did not have an abdominal ultrasound performed and intrahepatic/extrahepatic cholestasis could have therefore been missed and cannot be ruled out as a contributing factor.

Both hypocholesterolemia and hypercholesterolemia have been reported in the literarure in dogs with HA. In a recent review article compiling data from several case series, hypocholesterolemia was reported in 13% of dogs with “typical” (HHHA) and 73% of dogs with “atypical” (EEHA) [15], while hypercholesterolemia was reported in a older paper in 14% of dogs with “typical” HA (HHHA) [16]. By comparison, our study reported a prevalence of hypocholesterolemia higher in “typical” (HHHA) dogs (36%) but lower in “atypical” (EEHA) dogs (58%), while a similar prevalence of hypercholesterolemia was reported in “typical” (HHHA) dogs (13%). In a retrospective study including 220 dogs with primary HA and 5 dogs with secondary HA, the authors reported a median [Chol]_s_ of 201 mg/dL (range: 31–758) which was higher than the median [Chol]_s_ reported in our study (112 mg/dL, range: 31–309) [16]. Lower [Chol]_s_ has been reported in dogs with EEHA *versus* in dogs with HHHA [7]. A similar trend was observed in our study despite a lack of statistical significance after Bonferroni’s adjustment. Furthermore, our study reported a negative linear regression between the predictor variable [Chol]_s_ and the outcome variable Na/K among dogs with HHHA. One theory behind this may be that dogs with EEHA have a longer duration of clinical signs prior to presentation and subsequent diagnosis, including anorexia, and subsequent decreased fat absorption [7, 37]. Glucocorticoids are believed to be important in fat absorption and mobilization, and steatorrhea can occur in humans with HA [38]. This is further corroborated by the lower [Chol]_s_ found in HA dogs with [Alb]_s_ < 28 g/L compared to those with [Alb]_s_ ≥ 28 g/L, and by the positive linear regession between the predictor variable [Chol]_s_ and the outcome variable [Alb]_s_. Albumin serves as a marker of intestinal integrity and nutriment absorption; however, it is also a classic negative acute phase protein [39]. Both factors —intestinal integrity and the acute phase response— could contribute to the association of lower [Chol]_s_ with lower [Alb]_s_, with albumin potentially being lower in atypical cases due to a protracted disease course. Increased lipid utilization resulting from elevated [eACTH]_p_ may also play a role in cases of primary HA where there is no negative feedback occurring (Fig. 7) [40]. Another possible explanation for lower [Chol]_s_ in dogs with EEHA and low [Alb]_s_ is that some EEHA dogs can present with features of a secondary protein-losing enteropathy [41].

The main limitation of our study is due to its retrospective design, with a lack of standardization in diagnostic testing for endocrinopathies and other concurrent causes of dyslipidemia. Other contributors to dyslipidemia could have been missed due to: 1 – a lack of diagnostic testing (e.g., most patients diagnosed with hypothyroidism did not undergo biliary tree ultrasound); 2 – a lack of documentation of body condition score (mainly due to the retrospective design and the number of clinicians involved in the cases), since obesity has been identified as a cause of secondary hyperlipidemia in dogs [42–44] ; 3 – the inclusion of some breeds reported to have primary hypercholesterolemia [1], albeit they represented only 11/250 (4%) of included dogs; 4 – the incertitude that all serum samples were collected after a 12-hour fast when this information was missing in the medical database. Although every owner is instructed to fast their dog overnight prior to appointment (minimum 12 h), some patients presented through the emergency service (such as cases of DKA), and the duration of fasting was unknown in some cases. A recent study, however, showed that [Chol]_s_ was not significantly altered postprandially in 100 healthy dogs [45]. This finding is supported by the fact that postprandial conditions are more commonly associated with hypertriglyceridemia rather than hypercholesterolemia [1]. Therefore, if these findings in healthy dogs extrapolate to sick dogs, potential postprandial hypercholesterolemia would have not significantly interfered with the current results.

## Conclusion

In conclusion, hypercholesterolemia was very common in dogs with hypothyroidism (91%), DM (85%), or HAC (81%), and the prevalence of hypocholesterolemia in HA dogs was higher (45%) compared to previous reports. While not a revelatory finding, it is the first study directly comparing [Chol]_s_ in these 4 common endocrinopathies. Dogs with hypothyroidism displayed the highest median [Chol]_s_ when compared to all studied endocrinopathies. Additionally, median [Chol]_s_ was significantly higher in dogs with more severe hypothyroidism when defined as a lower [TT4]_s_ < 0.47 µg/dL vs. ≥ 0.47 µg/dL (i.e., < 6 nmol/L vs. ≥ 6 nmol/L), and in HAC dogs with [ALP]_s_ activity ≥ 1,000 U/L vs. < 1,000 U/L, but significantly lower in HA dogs with [Alb]_s_ < 28 g/L vs. ≥ 28 g/L. However, no trend was observed in the [Chol]_s_ in dogs with DM both with respect to the magnitude of hyperglycemia or with existence of secondary complications (i.e. DKA, HHS) at the time of diagnosis. While the diagnosis of endocrinopathies still necessitates specific endocrine tests along with corresponding clinical signs, serum cholesterol concentration can serve as a valuable tool to guide clinicians to suspect and diagnose certain endocrinopathies in dogs.

## Materials and methods

### Case selection

The medical records database of a referral teaching hospital (Veterinary Medical Center [VMC], Western College of Veterinary Medicine [WCVM], University of Saskatchewan, Canada) was retrospectively reviewed for dogs diagnosed with common endocrinopathies, namely hypothyroidism, DM, HAC, and HA, between the period of January 2011 and February 2022. The keywords “hypothyroidism/hypothyroid”, “diabetes/diabetic”, “hyperadrenocorticism/Cushing”, and hypoadrenocorticism/Addison” were used to search for the respective groups. To be included in each group, all dogs were required to exhibit clinical signs corresponding to the specified endocrinopathy, not have been previously diagnosed or treated for the specified endocrinopathy, and must have had a CBC and serum chemistry performed at the time of diagnosis. Additional criteria for the diagnosis of each endocrinopathy were applied as described below, including clinical signs, physical examination findings, routine bloodwork and diagnostic imaging parameters.

Hypothyroid dogs exhibited ≥ 1 compatible clinical signs (dermatologic abnormalities [symmetrical non-pruritic body alopecia, hyperpigmentation, hyperkeratosis, seborrhoea, hypertrichosis, facial myxoedema, poor wound healing, skin bruises, fading of coat color, failure to hair regrowth after clipping, superficial pyoderma, otitis externa, demodicosis], lethargy, exercise intolerance, obesity, unexplained weight gain, constipation, heat-seeking behaviors, sinus bradycardia, vestibular disease, polyneuropathy, corneal lipid deposits, and lipemic uveitis) along with decreased [TT4]_s_ (< 0.9 µg/dL or < 12 nmol/L) and increased [TSH]_s_ concentrations (> 0.58 ng/mL). Dogs with low [TT4]_s_ but normal [TSH]_s_ were included after review by an ACVIM-board-certified internist (ES) to determine their inclusion in the study based on other ancillary tests if available (e.g., serum free T4 concentration, serum thyroglobulin antibodies concentration, scintigraphy) and/or based on their response to treatment.

Dogs were diagnosed with DM according to the ALIVE criteria [46] if (1) a random (fasted or unfasted) blood glucose concentration was ≥ 200 mg/dL and they exhibited classic clinical signs of hyperglycemia (with no other plausible cause) or hyperglycemic crisis; or if (2) fasting blood glucose concentration was > 126 mg/dL, with or without clinical signs of hyperglcemia or hyperglycemic crisis, and persistent fasting hyperglycemia for > 24 h and/or increased glycated proteins was documented. Concurrent acute pancreatitis was recorded and diagnosed based on a combination of clinical signs, consistent ultrasonographic findings +/- an elevation in canine Pancreatic Lipase Immunoreactivity (cPLI) when available.

Dogs with HAC were included if they exhibited ≥ 1 compatible clinical signs (polyuria, polydipsia, a pot-bellied appearance, polyphagia, excessive panting, dermatologic abnormalities, muscle weakness or wasting), along with either an abnormal ACTH stimulation test (ACTHST) or a low-dose dexamethasone suppression test (LDDST) consistent with HAC. When needed, cases were reviewed by a ACVIM-board-certified internist (ES) to determine their inclusion in the study based on their signalment, clinical signs, bloodwork, other ancillary tests if available (e.g., abdominal ultrasound, brain or abdominal computed tomography scan, brain magnetic resonance imaging, necropsy), and response to treatment.

For HA, all included dogs exhibited ≥ 1 compatible clinical signs (lethargy, chronic or acute gastrointestinal signs [diarrhea, vomiting, regurgitations, melena, hematemesis], hypovolemia, hypotension, painful abdomen), and had an ACTHST performed, with a post-ACTH cortisol value < 2 ng/dL (< 55 nmol/L). Dogs with iatrogenic HA (such as mitotane-induced HA or adrenectomy) were excluded. Dogs were diagnosed with HHHA based on a consistent ACTHST and documentation of hyponatremia and/or hyperkalemia, while dogs were diagnosed with EEHA based on a consistent ACTHST and normal serum concentrations of potassium and sodium at the time of diagnosis.

Other exclusion criteria included: dogs with multiple endocrinopathies, extrahepatic biliary tract obstruction (EHBO), or receiving drugs reported to alter lipid metabolism (such as glucocorticoids, phenobarbital, stilbestrol, etc.) in the past 3 months prior to inclusion [1, 2].

### Medical records review

For all dogs with hypothyroidism, DM, HAC, or HA, medical records were reviewed for signalment (age, sex, breed), routine laboratory data (CBC, serum biochemistry, urinalysis), endocrine diagnostic testing, results of any diagnostic imaging when available, comorbidities and current medications. All diagnostic imaging studies were performed by Diplomates of the American College of Veterinary Radiology (ACVR) or a radiology resident under direct supervision of a board-certified radiologist.

### Statistical analyses

Statistical analyses and graphs were performed using a commercial software package (GraphPad PRISM10 software version 10.4.0, La Jolla, CA, USA). The normality of variables was tested with the Shapiro-Wilk W test. For two continuous, independent, normally distributed variables, the t-test was performed to compare means and standard deviations. For two continuous, independent, non-normally distributed variables, the Mann-Whitney Wilcoxon was performed to compare the medians. For more than 2 continuous independent non-normally distributed variables, the Kruskal-Wallis One-Way ANOVA test was performed, and the posthoc comparison was done using Dunn’s test with Bonferroni correction.

Effect size with 95% confidence intervals were provided for both mean and median differences. The differences between means were reported as exact differences while the difference between medians were reported as Hodges-Lehmann estimates. To calculate the confidence interval of the median difference as part of the Mann-Whitney Wilcoxon test, the software found the closest confidence level to 95% interval, and the precise confidence level (e.g. 95.3% in lieu of 95%) was reported.

Seven comparisons of [Chol]_s_ were performed in the following groups: hypothyroid dogs with [TT4]_s_ < 0.47 vs. [TT4]_s_ ≥ 0.47 µg/dL; dogs with uncomplicated DM vs. complicated DM (DKA or HHS); dogs with complicated DM with or without concurrent acute pancreatitis; DM dogs with serum blood glucose concentration < 450 vs. ≥ 450 mg/dL; HAC dogs with [ALP]_s_ < 1,000 vs. ≥ 1,000 U/L; HA dogs with [Alb]_s_ < 28 vs. ≥ 28 g/L; HA dogs with HHHA vs. EEHA. After Bonferroni’s adjustment, a *p*-value of < 0.0071 (i.e., < 0.05/7) was considered statistically significant. In dogs with DM, 6 serum chemistry parameters were compared between dogs with non-complicated DM or complicated DM (i.e., DKA or HHS). After Bonferroni’s adjustment, a *p*-value of < 0.0083 (i.e., < 0.05/6) was considered statistically significant. In dogs with HA, 9 serum chemistry parameters were compared between dogs with EEHA and HHHA. After Bonferroni’s adjustment, a *p*-value of < 0.0055 (i.e., < 0.05/9) was considered statistically significant.

For linear regressions, [Chol]_s_ was used as a predictor variable, and the tested outcome variables were [TT4]_s_, [TSH]_s_, serum blood glucose concentration, [ALP]_s_, post-ACTHST serum cortisol concentration, serum cortisol concentration at T8h after LDDST, [Alb]_s_ and [Na/K]_s_. The following required assumptions were tested: independent variables, independent and normally distributed residuals, homoscedasticity and linear relationship between the predictor and the outcome. The Cook-Weisberg test was used to confirm homoscedasticity. A *p*-value of < 0.05 was used to determine statistical significance.

For correlation analysis between [Chol]_s_ and [ALP]_s_ (non-parametric data sets), Spearman correlation coefficient (*r*_*s*_) was determined. A *p*-value of < 0.05 was used to determine statistical significance.

For other comparisons, a *p*-value of < 0.05 was used to determine statistical significance.

## Data Availability

The datasets used and/or analysed during the current study are available from the corresponding author on reasonable request.

## Abbreviations

ACTHST: Adrenocorticotropic hormone stimulation test
ADH: Adrenal-dependent hyperadrenocorticism
[Alb]_s_: Serum albumin concentration
CBC: Complete blood count
[Chol]_s_: Serum cholesterol concentration
cPLI: Canine pancreatic lipase immunoreactivity
DKA: Diabetic ketoacidosis
DM: Diabetes mellitus
[eACTH]_p_: Plasma endogenous adrenocorticotropic hormone concentration
EEHA: Eunatremic eukalemic hypoadrenocorticism
EHBO: Extrahepatic biliary tract obstruction
FFA: Free fatty acid
[fT4]_s_: Serum free thyroxin concentration
HA: Hypoadrenocorticism
HAC: Hyperadrenocorticism
HDDST: High-dose dexamethasone suppression test
HDL: High-density lipoproteins
HHHA: Hyponatremic and/or hyperkalemic hypoadrenocorticism
HSL: Hormone-sensitive lipase
LDDST: Low-dose dexamethasone suppression test
LDL: Low-density lipoproteins
LPL: Lipoprotein lipase
PDH: Pituitary-dependent hyperadrenocorticism
TG: Triglycerides
[TSH]_s_: Serum thyroid stimulating hormone (TSH) concentration
TSH: Thyroid stimulating hormone
[TT4]_s_: Serum total thyroxine concentration
